# C3 Compound Metabolism in the Thermoacidophilic Methanotroph *Methylacidiphilum fumariolicum* SolV

**DOI:** 10.1111/1758-2229.70129

**Published:** 2025-07-08

**Authors:** Changqing Liu, Arjan Pol, Stijn Peeters, Rob A. Schmitz, Theo A. van Alen, Lena J. Daumann, Huub J. M. Op den Camp, Wouter Versantvoort

**Affiliations:** ^1^ Department of Microbiology Radboud Institute for Biological and Environmental Sciences, Faculty of Science, Radboud University Nijmegen the Netherlands; ^2^ Department of Biotechnology Delft University of Technology Delft the Netherlands; ^3^ Chair of Bioinorganic Chemistry Heinrich‐Heine Universität Düsseldorf Germany

**Keywords:** 2‐propanol, acetone, methane, verrucomicrobial methanotroph

## Abstract

Terrestrial and oceanic geothermal areas emit substantial amounts of hydrocarbons in the form of methane and the short‐chain alkanes ethane and propane. Under hydrothermal conditions, these alkanes can also be oxidised to their respective alcohols and ketones, with a preference for the 2‐position. The thermoacidophilic verrucomicrobial methanotroph *Methylacidiphilum fumariolicum* SolV, isolated from the Solfatara volcano, was previously shown to oxidise methane as well as the short‐chain hydrocarbons propane and ethane. Here, we show the growth of strain SolV on the C3 compounds 2‐propanol and acetone with growth rates of 0.054 h^−1^ and 0.042 h^−1^, respectively. In contrast to methanotrophic growth (rate 0.07 h^−1^), growth was not dependent on CO_2_ or lanthanides. Respiration experiments on steady‐state continuous cultures showed an apparent affinity of 0.4 μM acetone and 5.4 μM 2‐propanol. Transcriptomic analysis of these cultures showed that a gene cluster including a novel acetone monooxygenase (PMO3), previously identified in the closely related species *Methylacidiphilum caldifontis*, was highly upregulated under growth on C3 substrates. These results support the versatile metabolism of verrucomicrobial methanotrophs. The conversion of other compounds besides methane can be important in view of the ecological relevance of methanotrophs.

## Graphical abstract

Terrestrial and oceanic geothermal areas emit substantial amounts of small hydrocarbons. The thermoacidophilic verrucomicrobial methanotroph *Methylacidiphilum fumariolicum* SolV, isolated from the Solfatara volcano, was shown to grow on 2‐propanol and acetone using steady‐state continuous cultures.

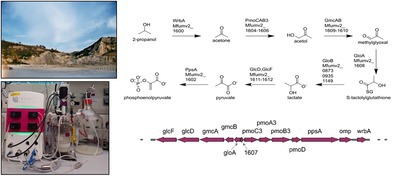

## 1 Introduction

Methane is a potent greenhouse gas and is emitted into the atmosphere from a wide variety of ecosystems, ranging from landfills to livestock and wetlands to geothermal areas (Dean et al. 2018; Houghton et al. 2019; Schaefer et al. 2016). Geothermal areas also emit substantial amounts of short‐chain alkanes (SCA) such as ethane (~2–4 Tg/y) and propane (~1–2.4 Tg/y), which contribute to photochemical pollution and ozone formation in the atmosphere (Capaccioni and Mangani 2001; Etiope and Ciccioli 2009; Etiope et al. 2013). Whereas the function of methanotrophic bacteria as biofilters for the mitigation of methane emission from geothermal areas to the atmosphere has been extensively studied (Dunfield et al. 2007; Islam et al. 2008; Pol et al. 2007; Schmitz et al. 2021; Sharp et al. 2014; van Teeseling et al. 2014), research on the role of microorganisms in the mitigation of SCA emissions from these ecosystems is scarce (Awala et al. 2021; Picone et al. 2020). Under hydrothermal conditions, in the presence of water, iron‐bearing minerals and aqueous sulfur, these SCAs are stepwise oxidised to their respective alkenes, alcohols and ketones, with a preference for the 2 positions (Seewald 2001).

The metabolism of methane and SCAs was initially thought to be performed by separate groups of microorganisms (Crombie and Murrell 2014), although earlier studies hinted at the ability of methanotrophs to oxidise ethane, propane and butane as well (Ashraf et al. 1994; Hamamura and Arp 2000; Hamamura et al. 1999; Kinnaman et al. 2007; Kniemeyer et al. 2007; Takahashi 1980). Growth on propane by a methanotroph was first described for the α‐proteobacterial methanotroph *Methylocella silvestris* (Crombie and Murrell 2014), utilising a soluble di‐iron propane monooxygenase (PrMMO). This strain was capable of growth on putative intermediates, such as 2‐propanol and acetone. Later, the ability of the verrucomicrobial methanotroph *Methylacidiphilum fumariolicum* SolV to co‐metabolise ethane and propane was shown, although growth solely on ethane or propane could not be demonstrated (Picone et al. 2020). No homologue of the PrMMO was identified in verrucomicrobial methanotrophs, but transcriptomics revealed the upregulation of the divergent particulate methane monooxygenase PMO3 in the presence of propane (Picone et al. 2020). Recently, two novel isolates, *Methylacidiphilum caldifontis* IT6 and *Methylacidiphilum infernorum* IT5, were shown to co‐metabolise propane as well. In addition, they were able to grow on a range of C3 compounds (2‐propanol, acetone, acetol) in the absence of methane (Awala et al. 2021). This study showed the role of PMO3 as a dedicated acetone monooxygenase, catalysing the formation of acetol.

PMO3 is part of a big gene cluster (13 genes) that was fully upregulated under C3‐dependent growth (Awala et al. 2021). This cluster also contains an oxidoreductase belonging to the GMC superfamily, known for its functional diversity (Sutzl et al. 2019). This GMC oxidoreductase has been postulated to oxidise both 2‐propanol to acetone and acetol to methylglyoxal (Awala et al. 2021; Bordel et al. 2020). The gene cluster further contains a lactoylglutathione lyase, lactate dehydrogenase and phosphoenolpyruvate (PEP) synthase that could convert methylglyoxal to pyruvate, which can enter the tricarboxylic acid (TCA) cycle, or be converted to phosphoenolpyruvate for gluconeogenesis. Thereby, this whole cluster would support growth on C3 substrates in verrucomicrobial methanotrophs (Awala et al. 2021).

Here the potential of *M. fumariolicum* SolV to metabolise and grow on the oxidised propane derivatives 2‐propanol and acetone was investigated through a combination of cultivation and respiration experiments. Steady‐state continuous chemostat cultures were established on 2‐propanol and acetone, after which their apparent kinetic parameters for propane, methane and their oxygenated derivatives were meticulously determined using respiratory experiments. These findings were complemented with transcriptomics to identify the metabolic pathway required for these growth adaptations.

## 2 Experimental Procedures

### 2.1 Microorganism and Medium Composition

The *M. fumariolicum* strain SolV used in this study was originally isolated from a mudpot sample taken at the Solfatara volcano (Pozzuoli, near Naples, Italy) (Pol et al. 2007). The cultivation medium was composed of 0.2 mM MgCl_2_·6 H_2_O, 0.2 mM CaCl_2_·2 H_2_O, 1 mM Na_2_SO_4_, 2 mM K_2_SO_4_, 4 mM (NH_4_)_2_SO_4_ and 1 mM NaH_2_PO_4_·H_2_O. A solution of trace elements was added, resulting in the following final concentrations: 1 μM NiCl_2_, CoCl_2_, MoO_4_Na_2_, ZnSO_4_ and CeCl_3_; 5 μM MnCl_2_ and FeSO_4_; 10 μM CuSO_4_ and 40–50 μM nitrilotriacetic acid (NTA). The pH of the medium was adjusted to 3.0 with 1 M H_2_SO_4_. To avoid precipitation, CaCl_2_·2H_2_O and the rest of the medium were autoclaved separately and mixed after cooling. The same medium composition was used in batch and continuous cultures unless stated otherwise.

### 2.2 Batch Cultivation

Batch culture experiments were performed in 120 mL serum bottles with 10 mL medium and a headspace containing air, 5% CO_2_ and 10% CH_4_. The bottles were closed with butyl rubber stoppers and capped with aluminium crimp caps. To test growth on 2‐propanol and acetone, a final concentration of 13 mM of these compounds was used instead of methane. To test growth on 1‐propanol and 1,2‐propanediol, either 2‐propanol‐grown or methane‐grown cells were transferred to medium containing 13 mM of 1‐propanol or 1,2‐propanediol. To test if strain SolV could oxidise propane, a 120 mL serum bottle was filled with 10 mL of new medium and 10 mL of biomass from a chemostat culture receiving 2‐propanol or acetone (see below) The headspace consisted of air, 2% propane and 10% CO_2_. The bottles were incubated in a shaking incubator (New Brunswick Inova 40) at 55°C shaking at 350 rpm except for the 2‐propanol and acetone cultures, which were shaken at 200 rpm. The effect of CO_2_ and lanthanides on the growth on 2propanol was investigated by growing 2‐propanol–adapted cells with or without 5% CO_2_ and with or without the addition of 1 μM LaCl_3_ to the growth medium. Bacterial growth was determined by measuring the increase in absorbance at 600 nm (OD_600_) in 1.5 mL disposable semimicro PS cuvettes (Brand, Wertheim, Germany) in a Cell Density meter model 40 (Fisher Scientific) or a Cary 60 spectrophotometer (Agilent Technologies, Santa Clara, CA, United States).

### 2.3 Chemostat Cultivation With Acetone and 2‐Propanol

Continuous cultivation was performed in 500 mL bioreactors (Applikon Delft, the Netherlands). The medium described above was supplemented with either acetone or 2‐propanol to obtain a final concentration of 26 mM. Parameter settings were the same for both bioreactors unless otherwise indicated. The bioreactor was operated at 55°C with stirring at 1000 rpm. Growth medium was supplied to the chemostat at a flow rate of 15 mL·h^−1^ (*D* = 0.0375 h^−1^) and maintained at 400 mL liquid volume by a peristaltic pump controlled by a level sensor. A gas supply of Ar/CO_2_ (95:5, v/v) was provided by mass flow controllers through a 0.2 μm sterile filter and sparged into the medium at a gas flow rate of 4 mL·min^−l^. Oxygen concentration was regulated at 20% air saturation by a separate mass flow controller. The pH was kept stable at pH 3 by titrating with 0.5 M NaOH or 0.5 M HCl. The biomass samples from both continuous cultures were used for RNA extraction, cell respiratory experiments and kinetic studies. At steady state with acetone or 2‐propanol as the limiting factor, the stable optical density of the culture was about 1.2 and 1.5, respectively.

### 2.4 Gas Chromatography

Methane and propane were measured by injecting a 100 μL headspace sample with a Hamilton glass syringe in a HP5890 gas chromatograph (Agilent, UAS) equipped with a Porapak Q column (1.8 m, ID 2 mm) and a flame ionisation detector.

Acetone, 2‐propanol and acetol were measured by gas chromatography using a HP‐INNOWAX column (30 m × 0.32 mm × 0.50 μm). Dinitrogen was used as carrier gas and the following conditions were applied: injector temperature of 200°C with a split ratio of 1:6, oven temperature of 110°C for 1 min and then increased with a gradient of 50°C/min to 250°C, held for 8 min. The quantification of 2‐propanol, acetone and acetol was carried out by comparison with standard aqueous solutions. Culture fluid (1–2 mL) was centrifuged at 15,000 × g for 10 min at room temperature. A 1 μL aliquot of the supernatant was injected into the gas chromatograph. For the measurement of 1‐propanol, propionaldehyde and propanoic acid, 1% formic acid was added before injection.

### 2.5 RNA Extraction, Transcriptomics and Analysis

Transcriptomics were performed according to a previously described protocol (Schmitz et al. 2023). For each triplicate (*n* = 3), 15 mL sample was collected from continuous cultures of strain SolV growing on either acetone or 2‐propanol on different days during the steady state. Cells were pelleted by centrifugation at 5000 × g for 10 min and immediately used for RNA isolation. Total RNA was isolated using the RiboPure RNA Purification Kit for bacteria (Thermo Fisher Scientific, Waltham, MA, USA) according to the manufacturer's protocol. RNA samples were treated with DNase to remove DNA contaminants. mRNA was enriched by removing ribosomal RNA from total RNA samples using the MICROBEexpress Bacterial mRNA Enrichment Kit (Thermo Fisher Scientific) according to the manufacturer's protocol. RNA concentrations of the extracted RNA were measured with the Qubitfluorometer and Qubit RNA HS Assay Kit (Thermo Fisher Scientific) and the RNA quality of both total RNA and enriched mRNA was determined using the Bioanalyzer system and the Agilent RNA 6000 Nano Kit (Agilent Technologies, Waldbronn, Germany) cDNA libraries were prepared from the rRNA‐depleted samples using TruSeq Stranded mRNA library prep kit (Illumina Inc., USA) according to the manufacturer's protocol. Libraries were diluted and sequenced using the Illumina Miseq sequencing machine (Illumina Inc.). Sequence reads were analysed using CLC Genomic Workbench software (version 12, Qiagen). Transcriptome reads were quality checked using FastQC (Andrews 2010) and subsequently trimmed by removal of 10 base pairs at the 5′ and 5 base pairs at the 3′ end of each read. Reads were mapped against the *M. fumariolicum* SolV genome (Anvar et al. 2014) using Bowtie2 (Langmead and Salzberg 2012). Mapped read counts per gene were determined using Rsubread (Liao et al. 2019) and fold changes and dispersion were estimated using DEseq2 (Love et al. 2014). Expression values were calculated as Transcripts Per Kilobase Million (TPM) to allow for easy comparison between samples. In addition to the samples on acetone and 2‐propanol collected in this study, transcriptomic data from a previous study on a continuous methane culture (Schmitz et al. 2023) were used for comparison. For analysing differential expression, a Wald test was used by DEseq2 to calculate adjusted *p*‐values. Differences in counts were considered to be significant if the basement was higher than 4, the log_2_‐fold change was higher than 0.5 and the adjusted *p*‐value was ≤ 0.05.

### 2.6 Cell Respiratory Experiment

Continuous 2‐propanol and acetone cultures were used to test substrate‐dependent oxygen consumption. These experiments were performed in a glass incubation chamber with a glass stirrer bar, as described previously (Schmitz et al. 2023) and fitted with an oxygen sensor spot (OXSP5, Pyroscience) coupled to a FireSting optical O_2_ meter (PyroScience), using 1 mL of whole‐cell suspensions of strain SolV. Pure oxygen was injected into the respiration chamber to obtain 50–100% air saturation, after which liquid substrate (acetone, acetol, methylglyoxal, methanol, 1‐propanol, 2‐propanol, 1,2‐propanediol) was injected into the same respiration chamber to reach a final concentration of 10 μM to 1 mM. The temperature and stirring rate in the respiration chamber were adjusted to 55°C and 1000 rpm, respectively. Rates were expressed as μmol O_2_ min^−1^ · g DW^−1^ and, when necessary, corrected for endogenous respiration. Varying concentrations of acetone (0–10 μM) and 2‐propanol (0–50 μM) were separately injected in the respiration chamber and used to determine Michaelis–Menten kinetics as described above.

## 3 Results

**FIGURE 1:**
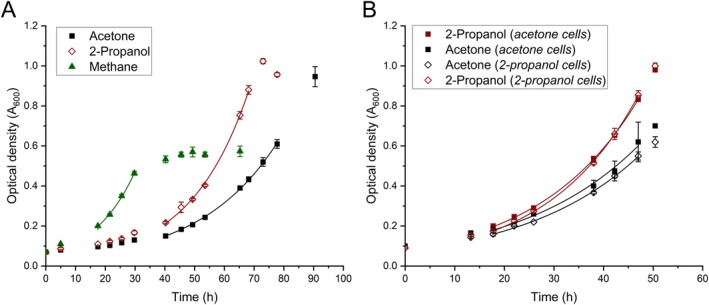
Growth of *M. fumariolicum* SolV on 2‐propanol, acetone and methane. (A) Cells from a batch culture on 10% CH_4_ were used to inoculate medium (10% transfer) containing different substrates: 10% CH_4_ (green closed triangle), 13 mM 2‐propanol (red open diamond) or 13.5 mM acetone (black closed square). Growth rates of 0.070, 0.051 and 0.038 h^−1^ were determined when growing on methane, 2‐propanol and acetone, respectively. (B) Acetone‐grown cells (closed squares) were used to inoculate (10% transfer) a medium containing 13 mM 2‐propanol (red closed square) or 13.5 mM acetone (black closed square), for which a growth rate of 0.049 and 0.041 h^−1^ was determined, respectively. 2‐Propanol‐grown cells (open diamonds) were used to inoculate (10% transfer) in a medium containing 13 mM 2‐propanol (red open diamond) or 13.5 mM acetone (black open diamond), for which a growth rate of 0.054 and 0.042 h^−1^ was determined respectively. The headspace of all cultures was supplemented with 5% v/v CO_2_. The OD_600_ values were plotted over time and an exponential fit (lines) was performed on the exponential phase to determine the growth rates. Data is represented as the mean with error bars representing standard deviation (*n* = 3).

*M. fumariolicum* SolV cells grown in batch on 10% CH_4_/5% CO_2_ were transferred to a medium without methane, containing either 13 mM 2‐propanol or 13.5 mM acetone with a 5% CO_2_ headspace as energy and carbon (Figure 1A). Compared to the control grown on 10% CH_4_/5% CO_2_ (μ = 0.070 h^−1^, doubling time of ~10 h), cells on 2‐propanol or acetone exhibited a lag phase of ~30 h before starting exponential growth, after which they reached growth rates of 0.051 h^−1^ (doubling time 13.6 h) and 0.038 h^−1^ (doubling time of 18.2 h), respectively. Methane was depleted after 30 h, resulting in the cells reaching stationary phase at an OD_600_ of ~0.5. When 2‐propanol and acetone‐grown cells were transferred (10%) into fresh medium, growth rates increased slightly to 0.054 h^−1^ and 0.041 h^−1^ for growth on 2‐propanol and acetone, respectively (Figure 1B). When acetone‐grown cells were transferred to a medium containing 2‐propanol a growth rate of 0.049 h^−1^ was observed. 2‐Propanol cells transferred to an acetone‐containing medium exhibited a growth rate of 0.042 h^−1^. Transferring acetone‐grown cells to a medium supplemented with 1‐propanol or 1,2 propanediol as the sole energy source did not result in any growth.

**FIGURE 2:**
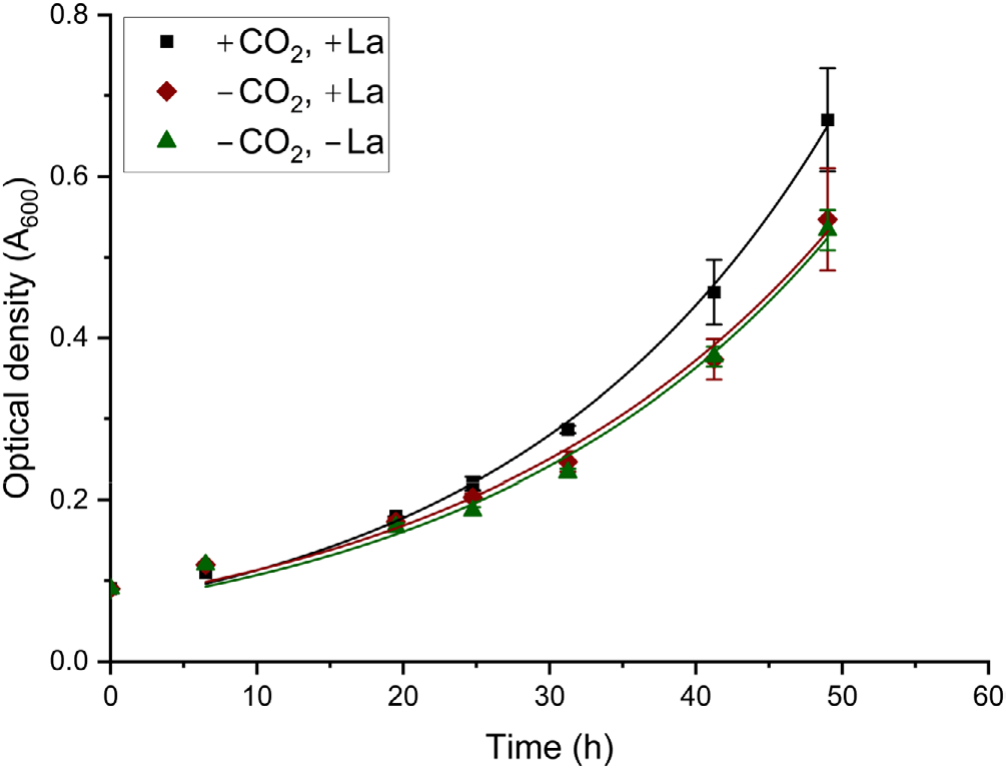
Influence of CO_2_ and lanthanum on the growth of M. fumariolicum SolV on 2‐propanol. Cells were inoculated in a medium containing 26 mM 2propanol/5% CO_2_ (black squares), 2‐propanol without CO_2_ (red diamonds) or 2‐propanol without CO_2_ and without lanthanides (green triangles), after an initial batch cultivation on 2‐propanol without CO_2_ and without lanthanides for 5 consecutive transfers to fresh medium with a 20 times dilution of the cells. The OD_600_ value was plotted over time and an exponential fit was performed on the exponential phase to determine the growth rates (μ). Data is represented as the mean with error bars representing standard deviation (*n* = 3).

Cells grown in batch on 2‐propanol/5% CO_2_ were transferred to culture media without lanthanides and without CO_2_, for 5 consecutive growth cycles to OD ~1, to deplete internal La storages. Subsequently, growth on 2‐propanol with/without lanthanum (1 μM LaCl_3_) and with/without CO_2_ were compared (Figure 2 and Supplementary Figure S1) in two separate experiments. The mean growth rates were 0.047 h^−1^, 0.043 h^−1^ and 0.044 h^−1^ for cells grown with CO_2_ and La, without CO_2_ with La and without CO_2_ and La, respectively. Cells grown on 2‐propanol with CO_2_ seemed to grow slightly faster than cells without CO_2_, but a one‐way ANOVA analysis with a Tukey test to factor in multiple comparisons showed this difference was not significant (*p* = 0.33 for +CO_2_/+La vs. CO_2_/+La and *p* = 0.41 for +CO_2_/+La vs. −CO_2_/−La). The removal of lanthanum from the growth medium did not have an effect on the growth of SolV cells on 2‐propanol (*p* = 0.97 for −CO_2_/+La vs. −CO_2_/−La).

Since propane was also readily converted, the potential to grow on propane was examined for both 2‐propanol and acetone‐grown cells in batch cultures. For both, an initial consumption of propane was observed, which halted after ~50 h. No clear increase in OD values for either culture could be observed (Supplementary Figure S2), indicating propane could not be used as a sole substrate. The conversion of propane as a co‐substrate in the environment might offer some additional energy, which could support persistence but is insufficient to support growth.

### 3.1 Chemostat Cultivation on Acetone and 2‐Propanol and Respiratory Analysis

Two continuous chemostat cultures of *M. fumariolicum* SolV with either 26 mM 2‐propanol or 26 mM acetone as the sole energy source were established. Under substrate limitation, these cultures reached steady state (i.e., growth rate equals dilution rate) at stable OD_600_ values of 1.5 and 1.2 for 2‐propanol and acetone, respectively. Cells from both cultures were used to analyse the respiration rate on varying one‐and three‐carbon compounds (Table 1). Compared to cells grown on 2‐propanol, cells grown on acetone showed faster consumption of all tested compounds apart from 2‐propanol itself. Both cultures also respired methane, methanol and 1‐propanol, but did not respire 1,2‐propanediol.

**TABLE 1:** Substrate‐dependent O_2_ consumption rates (V) using whole SolV cells from chemostat cultures grown on either acetone or 2‐propanol.

Substrate	V acetone chemostat cells	V 2‐propanol chemostat cells
	(μmol O_2_ · min^−1^ · g DW^−1^)	(μmol O_2_ · min^−1^ · g DW^−1^)
Acetone	93.7 ± 3.1	87.3 ± 0.4
Acetol	118.5 ± 0.2	88.7 ± 5.3
Methylglyoxal	122.9 ± 4.2	40.5 ± 0
Methanol	126.7 ± 0.7	104 ± 0.7
1‐Propanol	62.2 ± 0.4	38.8 ± 0.4
2‐Propanol	51.5 ± 2.2	96.8 ± 2.5
1,2‐Propanediol	n.d.	b.d.l.

*Note:* Data are expressed as the mean of three biological replicates with standard deviation. b.d.l. = below detection limit (< 0.1). n.d. = not determined. All rates (V) are in μmol O_2_ · min^−1^ · g DW^−1^ with 1 mM liquid substrates (acetone, acetol, methylglyoxal, methanol, 1‐propanol, 2‐propanol, 1,2‐propanediol).

**FIGURE 3:**
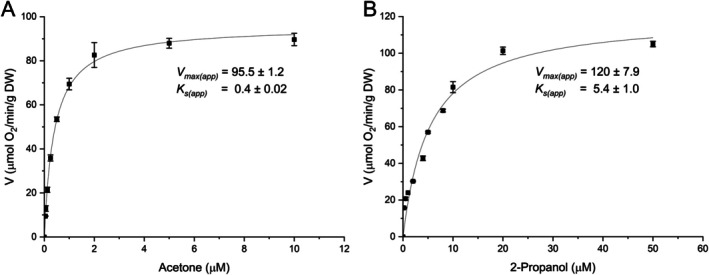
Oxygen consumption of acetone‐grown cells on varying acetone (**A**) and 2‐propanol‐grown cells on varying 2‐propanol **(B)** concentrations. Black lines represent a curve fit using Michaelis–Menten kinetics. Data is represented as the mean with error bars indicating standard deviation (*n* = 3).

To determine the maximum respiration rate and affinity for acetone and 2‐propanol, respiratory kinetics were measured using 0–10 μM acetone (Figure 3A) and 0–50 μM 2‐propanol (Figure 3B), with SolV cells grown on these respective substrates. Michaelis–Menten fits of the oxygen consumption data showed a maximum respiration rate of 95.5 ± 1.2 μmol O_2_·min^−1^·g DW^−1^ on acetone, with an apparent affinity of 0.4 ± 0.02 μM acetone. For 2‐propanol, the maximum respiration rate of 120 ± 7.9 μmol O_2_·min^−1^·g DW^−1^ was determined, with an apparent affinity of 5.4 ± 1.0 μM for 2‐propanol.

### 3.2 Transcriptomic Analysis on 2‐Propanol & Acetone

**FIGURE 4:**
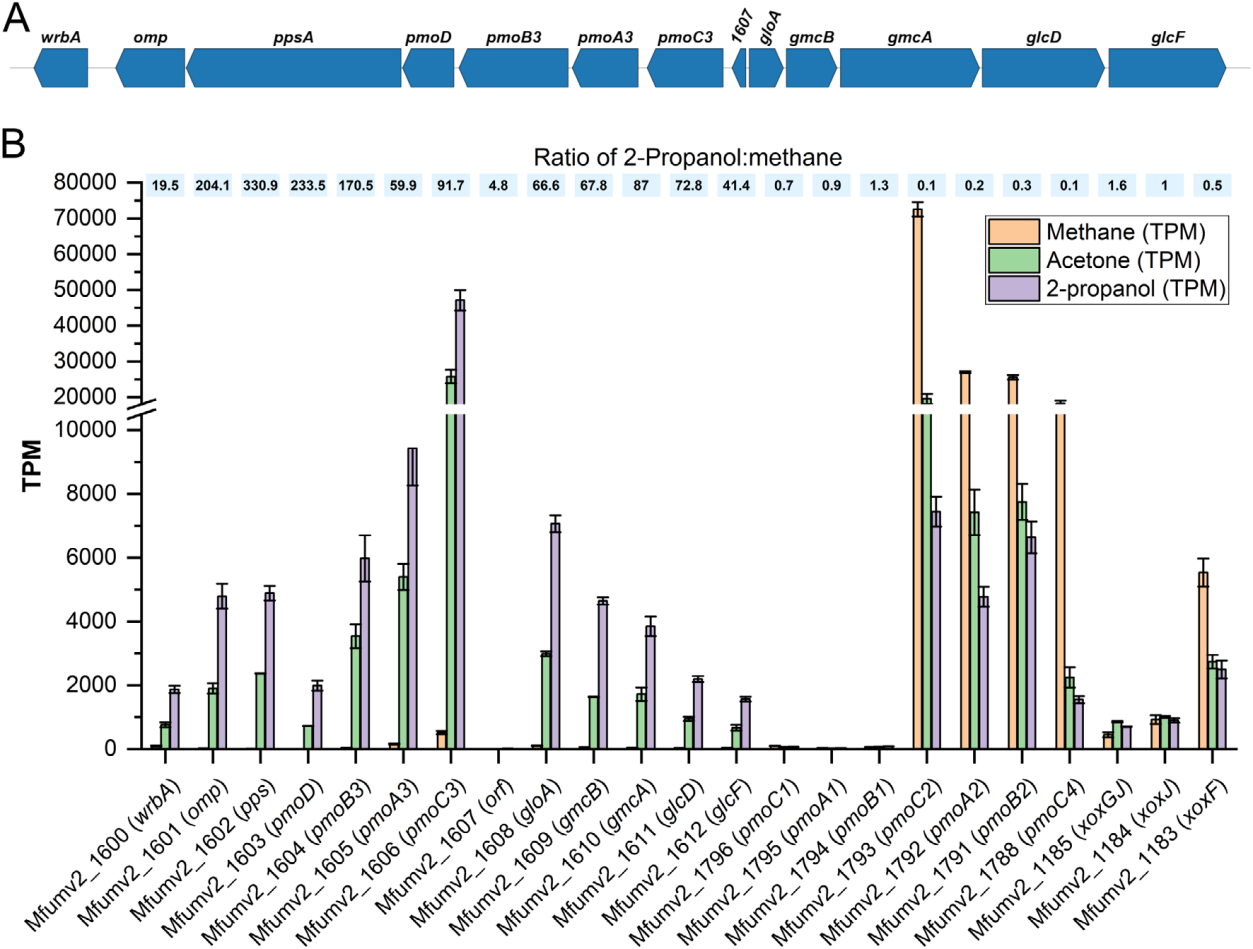
(A) Gene cluster Mfumv2_1600–1612 proposed to be involved in C3 metabolism of *M. fumariolicum* SolV. (B) Transcriptome analysis (Transcripts per Kilobase Million (TPM)) of SolV grown on 2‐propanol (purple), acetone (green) and methane (orange) of selected genes (putatively) involved in C1 and C3 metabolism. Data is represented by the mean with error bars indicating standard deviation (*n* = 3).

To investigate the metabolic pathways responsible for 2‐propanol and acetone conversion in *M. fumariolicum* SolV, RNAseq was performed on steady‐state chemostat cultures utilising 2‐propanol and acetone for growth, respectively. These data were compared with previous RNAseq data on a methane‐grown steady‐state chemostat culture (Schmitz et al. 2023) to determine up/downregulation of specific genes. Several genes were significantly upregulated (log_2_FC ≥ 2 and padj ≤ 0.5) in cells growing on 2‐propanol/acetone, compared to methane (Supplementary Table S1). Amongst these genes, 20 were annotated as unknown proteins and transposases, whereas 35 genes involved in major metabolic pathways were identified (Supplementary Table S2). The most striking difference was observed in a gene cluster (*Mfumv2_1600–1612*, Figure 4) containing the *pmoCAB3* operon (*Mfumv2_1604–1606*). This cluster is barely expressed with methane as an energy source (8–500 TPM), but highly expressed under 2‐propanol/acetone growth (1500–47,000 TPM; 20‐330‐fold upregulation). Besides a copy of pMMO, this cluster contains genes encoding a putative alcohol dehydrogenase (w*rba1*, *Mfumv2_1600*), an unknown beta‐barrel containing membrane protein (*Mfumv2_1601*), PEP synthetase (*ppsA*, *Mfumv2_1602*), lactoylglutathione lyase (glyoxalase I, *gloA*, *Mfumv2_1606*), GMC‐type oxidoreductase (*gmcAB*, *Mfumv2_1609–1610*) and D‐lactate dehydrogenase (*glcF* and *glcD, Mfumv2_1611–1612*). These enzymes could be involved in the downstream pathway of acetone/2‐propanol oxidation. In addition, a putative particulate methane monooxygenase subunit D (*pmoD*, *Mfumv2_1603*) is part of this highly upregulated gene cluster (1987 TMP, 233‐fold upregulation). PmoD is a membrane protein with a C‐terminal transmembrane helix with the proposed function as a transport protein. Recently, direct evidence for the involvement of PmoD in acetone oxidation was described (Chau et al. 2022). The PmoD encoding gene from strain IT6 was heterologously expressed in *Methylomonas* sp. DH‐1. The coupling with its endogenous pMMO resulted in a boost in acetol production from acetone by cell suspensions. However, the specific mechanism by which PmoD assists the pMMO3 complex in acetone oxidation remains elusive.

The pmoCAB2 operon was significantly downregulated under 2‐propanol/acetone growth (4700–7400 TPM), compared to methane (25500–72,500 TPM, 4‐10‐fold downregulated), as was the Ln‐dependent XoxF methanol dehydrogenase (5500 to 2500 TMP, 2‐fold downregulated). Strikingly, *xoxGJ*, encoding the electron acceptor for MDH, was not down‐, but upregulated under 2‐propanol/acetone growth, compared to methane (440 to 700 TPM, 1.5‐fold upregulated).

## 4 Discussion

Here we show that *M. fumariolicum* SolV can metabolise and grow on the C3 compounds 2‐propanol and acetone, as previously shown for other verrucomicrobial methanotrophs *Methylacidiphilum* sp. IT5 and IT6 (Awala et al. 2021). After adaptation, strain SolV showed growth rates of 0.054 and 0.042 h^−1^ on 2‐propanol and acetone, respectively, compared to 0.042 and 0.039 h^−1^ observed for strain IT6 (Awala et al. 2021). Oxidation of 2‐propanol provides two extra electrons compared to acetone, which, according to modelling studies, would generate a higher flux through the terminal oxidase, supporting a higher growth rate (Saldivar et al. 2024). In addition to previous work (Awala et al. 2021), we obtained stable steady‐state chemostat cultures on these substrates. Growth was not dependent on the presence of CO_2_, indicating that, contrary to growth on CH_4_, another carbon assimilation pathway than the CBB pathway was active. Transcriptome experiments, using cells from the chemostat cultures, showed the upregulation of 10 genes encoding for TCA cycle enzymes (2.5‐10‐fold upregulation), allowing for heterotrophic growth on C3 compounds, as was observed before (Awala et al. 2021). In contrast to strain IT6, the addition of CO_2_ did have a positive growth effect on SolV. Modelling studies on strain Pic showed that under C3 growth, regeneration of NAD(P)H to NAD(P)^+^ is a limiting factor in their metabolism (Saldivar et al. 2024). Running the CBB cycle might offer an additional electron sink, allowing for a higher rate of substrate turnover. Alternatively, PEP carboxylase (*Mfumv2_2035*) is also roughly 2‐fold upregulated under C3 growth. Combined with the upregulation of PEP synthetase (*Mfumv2_1602*, *ppsA*) (14 to 4900 TPM, 330‐fold upregulation), a higher CO_2_ concentration at the start of cultivation might offer a higher flux of CO_2_‐dependent PEP conversion to oxaloacetate.

The absence or presence of La, the metal cofactor of XoxF‐type MDH (Fitriyanto et al. 2011; Hibi et al. 2011), did not influence the growth of strain SolV on C3 compounds. Transcriptome analysis showed strong downregulation of the *xoxF* gene and respiration rates on methanol for acetone (114 ± 0.6 μmol O_2_ · min^−1^ · g DW^−1^) and 2‐propanol grown cells (93.6 ± 0.6 μmol O_2_ · min^−1^ · g DW^−1^) are considerably lower than for cells grown on methane (311 ± 22 μmol O_2_ · min^−1^ · g DW^−1^) (Schmitz et al. 2023), despite the presence of lanthanides in the growth medium. Finally, XoxF from SolV has been shown to oxidise 1‐propanol, but not 2‐propanol (Pol et al. 2014). Combined, this shows that the XoxF‐type MDH is not involved in C3 metabolism. This is in contrast to *Methylocella silvestris* BL2, where growth on 2‐propanol and propane was positively affected by the addition of lanthanides (Crombie 2022). Here, the gene encoding XoxF5 was upregulated in the presence of Ln. XoxF5 has a broader substrate range than XoxF2 from SolV and is capable of 2‐propanol oxidation (Fitriyanto et al. 2011; Huang et al. 2018). The strict dependence of SolV on Ln for its C1 metabolism, due to the absence of the Ca‐dependent MDH, has hampered the investigation of its molecular mechanism for lanthanide uptake. The ability to grow SolV in the absence of Ln on C3 compounds opens up the possibility to examine the effect of Ln addition on gene transcription. Thereby, the molecular mechanism of lanthanide uptake and gene regulation in these verrucomicrobial methanotrophs can be further investigated.

In contrast to XoxF, the *xoxGJ* gene, encoding for cytochrome *c*_*GJ*_, the dedicated electron acceptor of XoxF‐type MDH (Versantvoort et al. 2019) was slightly upregulated under C3 growth (1.5‐fold). It is therefore tempting to speculate that this cytochrome *c*_*GJ*_ can function as an electron acceptor of an oxidation step in 2‐propanol/acetone metabolism as well. However, this upregulation of *xoxGJ* was not observed for strain IT6 (Awala et al. 2021), further experiments are required to test this.

The *mfumv2_1600–1612* gene cluster, implicated in C3 metabolism (Awala et al. 2021), was upregulated 20–330‐fold under 2‐propanol and acetone growth. The transcriptome data of SolV, combined with physiological experiments, further corroborate the role of pMMO3 as acetone monooxygenase (Awala et al. 2021). GMC oxidoreductase has been shown capable of acetol conversion to methylglyoxal in *M. sylvestris* BL2 (Bordel et al. 2020) and was also significantly upregulated in SolV. It was also suggested to oxidise 2‐propanol to acetone in strain IT6 (Awala et al. 2021), although this activity is not biochemically validated. SolV also encodes for another potential alcohol dehydrogenase near this upregulated gene cluster (*Mfumv2_1600*, *wrbA*), which is 2.5 times upregulated between 2‐propanol and acetone‐grown cells. Combined with the observations that the growth of SolV on 2‐propanol was slower for cells previously grown on acetone than for cells previously grown on 2‐propanol, and respiration rates on 2‐propanol for 2‐propanol cells were double that of acetone cells, a dual function for GMC oxidoreductase seems unlikely. An alternative alcohol dehydrogenase such as *wrbA* could encode for a dedicated 2‐propanol dehydrogenase.

**FIGURE 5:**
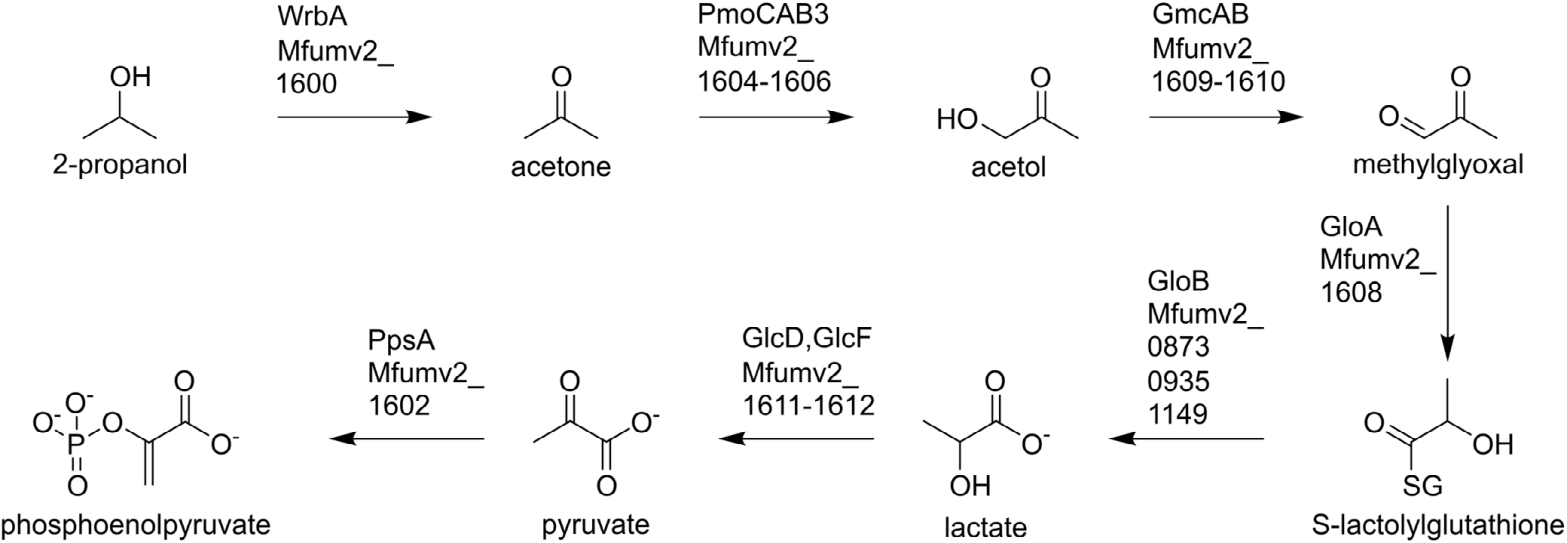
Pathway of 2‐propanol conversion to phosphoenolpyruvate in *Methylacidiphilum* spp. The genes encoding the enzymes catalysing the respective reactions are given above each arrow, with their corresponding identifier in the genome of *M. fumariolicum* SolV (Anvar et al. 2014).

SolV further contains a glyoxylase system consisting of glyoxylase I (*gloA*, *Mfumv2_1608*) and three copies of glyoxylase II (*gloB*, Mfumv2_0873/0935/1149) to convert methylglyoxal to lactate using thiol/glutathione as a cosubstrate (Suttisansanee and Honek 2011). Lactate can be oxidised to pyruvate by lactate dehydrogenase (*glcD, Mfumv2_1611*; *glcF*, *Mfumv2_1612*). This pyruvate can enter the TCA cycle or be converted to PEP by PEP synthetase. PEP can either be used for gluconeogenesis or enter the TCA cycle at the level of oxaloacetate (see above). These genes are also part of the *mfumv2_1600–1612* gene cluster upregulated under C3 growth. Combined, this gives SolV the complete genetic contents for the stepwise conversion of and growth on 2‐propanol (Figure 5) and upregulates these genes under 2‐propanol and acetone growth.

Methane respiration in SolV cells grown on methane is 302 ± 9 μmol O_2_ · min^−1^ · g DW^−1^ (Schmitz et al. 2023). Acetone and 2‐propanol respiration of chemostat cells grown on these substrates was 93.7 ± 3.1 μmol O_2_ · min^−1^ · g DW^−1^ and 96.8 ± 2.5 μmol O_2_ · min^−1^ · g DW^−1^, respectively, Table 1). Previously, propane conversion for SolV cells grown on a mixture of propane/CH_3_OH was reported to be 2.4 μmol O_2_^−1^· min · g DW^−1^ (Picone et al. 2020). Under propane/CH_3_OH, the pMMO1 encoding operon was the most transcribed pMMO, and slight upregulation of pMMO3 was also observed (Picone et al. 2020), although not at the levels seen under 2‐propanol/acetone growth. Conversely, for strain IT6, propane conversion was not observed for acetone‐grown cells, but only for methane‐grown cells (3.6 nmol propane · min ·^−1^ mg DW^−1^, (Awala et al. 2021). In SolV, pMMO2 is highly expressed under oxygen replete (18% oxygen saturation) conditions (RPKM values 10.9 × 10^3^ to 45 × 10^3^), whereas pMMO2&3 are hardly expressed (RPKM 21–253). Under oxygen limitation (0.5%–0.03% oxygen saturation) pMMO1 is highest expressed (RPKM values 4.1 × 10^3^ to 25 × 10^3^) and pMMO2 is 40‐fold downregulated compared to oxygen‐replete conditions, whereas pMMO3 is unaffected by a change in oxygen concentration (Khadem et al. 2012). Since the exact mechanism of pMMO is not yet well understood, it is difficult to pinpoint differences in pMMO sequences to their differences in observed activity, although the expression data support pMMO3 to be an acetone monooxygenase.

Growth of strain SolV, on propane alone was not observed, just like previously reported (Picone et al. 2020), and also for strain IT6 (Awala et al. 2021). Since SolV can grow properly on 2‐propanol, the main product of propane oxidation by pMMO is most likely 1‐propanol. This can be further oxidised to 1‐propanaldehyde and propanoate by XoxF‐MDH (Pol et al. 2014). SolV lacks the enzymes required to further degrade these toxic compounds, which prevents growth. In contrast to the verrucomicrobial methanotrophs, *Methylocella silvestris* and *Methylovirgula thiovorans*, belonging to the α‐proteobacteria, were observed to grow simultaneously on methane and propane (Crombie and Murrell 2014; Gwak et al. 2022). These two strains are capable of oxidising propane using the soluble di‐iron methane monooxygenase (sMMO) or the soluble di‐iron propane monooxygenase (PrMMO) (Bordel et al. 2020; Crombie and Murrell 2014; Gwak et al. 2022). Soluble di‐iron monooxygenases are key enzymes in the bacterial oxidation of gaseous hydrocarbons, including alkanes, alkenes, alcohols, ethers, alicyclics, aromatics, and chlorinated organic compounds (Banerjee et al. 2019), but are not present in the genomes of verrucomicrobial methanotrophs, which only contain copies of pMMO.

In summary, we show the capability of *M. fumariolicum* SolV to grow on C3 compounds 2‐propanol and acetone in batch and stable steady‐state chemostats. Detailed kinetic parameters for both 2‐propanol and acetone steady state cultures for a wide variety of C3 substrates and intermediates were determined. Transcriptomics corroborated the upregulation of a gene cluster responsible for C3 growth, including the novel acetone monooxygenase (Awala et al. 2021). This further highlights the versatile role of verrucomicrobial methanotrophs in the conversion of various substrates in their acidic geothermal habitat (Awala et al. 2021; Schmitz et al. 2021).

## Author Contributions

**Changqing Liu:** conceptualization, investigation, formal analysis, methodology, writing – original draft. **Arjan Pol:** conceptualization, methodology, investigation, writing – review and editing, supervision. **Stijn Peeters:** investigation, methodology, writing – review and editing. **Rob A. Schmitz:** investigation, writing – original draft. **Theo A. van Alen:** investigation, methodology, writing – review and editing. **Lena J. Daumann:** conceptualization, investigation, methodology, writing – review and editing, supervision. **Huub J. M. Op den Camp:** conceptualization, investigation, funding acquisition, methodology, writing – original draft, project administration, supervision. **Wouter Versantvoort:** conceptualization, investigation, writing – original draft.

## Ethics Statement

The authors have nothing to report.

## Consent

The authors have nothing to report.

## Conflicts of Interest

The authors declare no conflicts of interest.

## Supporting information


**Data S1.** Supporting Information.

**Figure S1.** | Effect of CO_2_ and lanthanides on the growth of M. fumariolicum SolV.
**Figure S2.** | Propane (grey diamonds) conversion and optical density (black squares) of *M. fumariolicum* SolV cells initially grown on (A) 2‐propanol and (B) acetone.

**Table S1.** Comparison of expression levels (RNAseq, full genome) of three different growth conditions Values are expressed as TPM with standard deviation (SD, *n* = 3).

**Table S2.** Comparison of expression levels (RNAseq) of enzymes of key metabolic pathways of three different growth conditions Values are expressed as TPM with standard deviation (SD, *n* = 3) and differential expression (fold change).

## Data Availability

The data that support the findings of this study are openly available in NCBI BioProject database at https://www.ncbi.nlm.nih.gov/bioproject/, reference number PRJNA766544.

## References

[emi470129-bib-0001] Andrews, S. 2010. “FastQC: A Quality Control Tool for High Troughput Sequence Data.” http://www.bioinformatics.babraham.ac.uk/projects/fastqc/.

[emi470129-bib-0002] Anvar, S. Y. , J. Frank , A. Pol , et al. 2014. “The Genomic Landscape of the Verrucomicrobial Methanotroph Methylacidiphilum Fumariolicum SolV.” BMC Genomics 15, no. 1: 914. 10.1186/1471-2164-15-914. 25331649 PMC4210602

[emi470129-bib-0003] Ashraf, W. , A. Mihdhir , and J. C. Murrell . 1994. “Bacterial Oxidation of Propane.” FEMS Microbiology Letters 122, no. 1–2: 1–6. 10.1111/j.1574-6968.1994.tb07134.x. 7958761

[emi470129-bib-0004] Awala, S. I. , J. H. Gwak , Y. M. Kim , et al. 2021. “Verrucomicrobial Methanotrophs Grow on Diverse C3 Compounds and Use a Homolog of Particulate Methane Monooxygenase to Oxidize Acetone.” ISME Journal 15, no. 12: 3636–3647. 10.1038/s41396-021-01037-2. 34158629 PMC8630023

[emi470129-bib-0005] Banerjee, R. , J. C. Jones , and J. D. Lipscomb . 2019. “Soluble Methane Monooxygenase.” Annual Review of Biochemistry 88, no. 1: 409–431. 10.1146/annurev-biochem-013118-111529. 30633550

[emi470129-bib-0006] Bordel, S. , A. T. Crombie , R. Munoz , and J. C. Murrell . 2020. “Genome Scale Metabolic Model of the Versatile Methanotroph *Methylocella silvestris* .” Microbial Cell Factories 19, no. 1: 144. 10.1186/s12934-020-01395-0. 32677952 PMC7364539

[emi470129-bib-0007] Capaccioni, B. , and F. Mangani . 2001. “Monitoring of Active but Quiescent Volcanoes Using Light Hydrocarbon Distribution in Volcanic Gases: The Results of 4 Years of Discontinuous Monitoring in the Campi Flegrei (Italy).” Earth and Planetary Science Letters 188, no. 3–4: 543–555. 10.1016/S0012-821x(01)00338-7.

[emi470129-bib-0008] Chau, T. H. T. , A. D. Nguyen , and E. Y. Lee . 2022. “Boosting the Acetol Production in Methanotrophic Biocatalyst Methylomonas sp. DH‐1 by the Coupling Activity of Heteroexpressed Novel Protein PmoD With Endogenous Particulate Methane Monooxygenase.” Biotechnology for Biofuels and Bioproducts 15, no. 1: 7. 10.1186/s13068-022-02105-1. 35418298 PMC8764830

[emi470129-bib-0009] Crombie, A. T. 2022. “The Effect of Lanthanum on Growth and Gene Expression in a Facultative Methanotroph.” Environmental Microbiology 24, no. 2: 596–613. 10.1111/1462-2920.15685. 34320271 PMC9291206

[emi470129-bib-0010] Crombie, A. T. , and J. C. Murrell . 2014. “Trace‐Gas Metabolic Versatility of the Facultative Methanotroph *Methylocella silvestris* .” Nature 510, no. 7503: 148–151. 10.1038/nature13192. 24776799

[emi470129-bib-0011] Dean, J. F. , J. J. Middelburg , T. Röckmann , et al. 2018. “Methane Feedbacks to the Global Climate System in a Warmer World.” Reviews of Geophysics 56, no. 1: 207–250. 10.1002/2017rg000559.

[emi470129-bib-0012] Dunfield, P. F. , A. Yuryev , P. Senin , et al. 2007. “Methane Oxidation by an Extremely Acidophilic Bacterium of the Phylum Verrucomicrobia.” Nature 450, no. 7171: 879–882. 10.1038/nature06411. 18004300

[emi470129-bib-0013] Etiope, G. , and P. Ciccioli . 2009. “Earth's Degassing: A Missing Ethane and Propane Source.” Science 323, no. 5913: 478. 10.1126/science.1165904. 19164741

[emi470129-bib-0014] Etiope, G. , A. Drobniak , and A. Schimmelmann . 2013. “Natural Seepage of Shale Gas and the Origin of “Eternal Flames” in the Northern Appalachian Basin, USA.” Marine and Petroleum Geology 43: 178–186. 10.1016/j.marpetgeo.2013.02.009.

[emi470129-bib-0015] Fitriyanto, N. A. , M. Fushimi , M. Matsunaga , A. Pertiwiningrum , T. Iwama , and K. Kawai . 2011. “Molecular Structure and Gene Analysis of Ce3+ −Induced Methanol Dehydrogenase of Bradyrhizobium sp. MAFF211645.” Journal of Bioscience and Bioengineering 111, no. 6: 613–617. 10.1016/j.jbiosc.2011.01.015. 21334970

[emi470129-bib-0016] Gwak, J. H. , S. I. Awala , N. L. Nguyen , et al. 2022. “Sulfur and Methane Oxidation by a Single Microorganism.” Proceedings of the National Academy of Sciences of the United States of America 119, no. 32: e2114799119. 10.1073/pnas.2114799119. 35914169 PMC9371685

[emi470129-bib-0017] Hamamura, N. , and D. J. Arp . 2000. “Isolation and Characterization of Alkane‐Utilizing Nocardioides sp. Strain CF8.” FEMS Microbiology Letters 186, no. 1: 21–26. 10.1111/j.1574-6968.2000.tb09076.x. 10779707

[emi470129-bib-0018] Hamamura, N. , R. T. Storfa , L. Semprini , and D. J. Arp . 1999. “Diversity in Butane Monooxygenases Among Butane‐Grown Bacteria.” Applied and Environmental Microbiology 65, no. 10: 4586–4593. 10.1128/AEM.65.10.4586-4593.1999. 10508093 PMC91611

[emi470129-bib-0019] Hibi, Y. , K. Asai , H. Arafuka , M. Hamajima , T. Iwama , and K. Kawai . 2011. “Molecular Structure of La3+−Induced Methanol Dehydrogenase‐Like Protein in *Methylobacterium radiotolerans* .” Journal of Bioscience and Bioengeneering 111, no. 5: 547–549. 10.1016/j.jbiosc.2010.12.017. 21256798

[emi470129-bib-0020] Houghton, K. M. , C. R. Carere , M. B. Stott , and I. R. McDonald . 2019. “Thermophilic Methanotrophs: In Hot Pursuit.” FEMS Microbiology Ecology 95, no. 11: fiz158. 10.1093/femsec/fiz158. 31374570

[emi470129-bib-0021] Huang, J. , Z. Yu , and L. Chistoserdova . 2018. “Lanthanide‐Dependent Methanol Dehydrogenases of XoxF4 and XoxF5 Clades Are Differentially Distributed Among Methylotrophic Bacteria and They Reveal Different Biochemical Properties.” Frontiers in Microbiology 9, no. 1664‐302X: 1366. 10.3389/fmicb.2018.01366. 29997591 PMC6028718

[emi470129-bib-0022] Islam, T. , S. Jensen , L. J. Reigstad , O. Larsen , and N. K. Birkeland . 2008. “Methane Oxidation at 55 Degrees C and pH 2 by a Thermoacidophilic Bacterium Belonging to the Verrucomicrobia Phylum.” Proceedings of the National Acadademy of Sciences of the United States of America 105, no. 1: 300–304. 10.1073/pnas.0704162105. PMC2224206 18172218

[emi470129-bib-0023] Khadem, A. F. , A. Pol , A. S. Wieczorek , M. S. M. Jetten , and H. J. Op den Camp . 2012. “Metabolic Regulation of “Ca. Methylacidiphilum Fumariolicum” SolV Cells Grown Under Different Nitrogen and Oxygen Limitations.” Frontiers in Microbiology 3: 266. 10.3389/fmicb.2012.00266. 22848206 PMC3404531

[emi470129-bib-0024] Kinnaman, F. S. , D. L. Valentine , and S. C. Tyler . 2007. “Carbon and Hydrogen Isotope Fractionation Associated With the Aerobic Microbial Oxidation of Methane, Ethane, Propane and Butane.” Geochimica et Cosmochimica Acta 71, no. 2: 271–283. 10.1016/j.gca.2006.09.007.

[emi470129-bib-0025] Kniemeyer, O. , F. Musat , S. M. Sievert , et al. 2007. “Anaerobic Oxidation of Short‐Chain Hydrocarbons by Marine Sulphate‐Reducing Bacteria.” Nature 449, no. 7164: 898–901. 10.1038/nature06200. 17882164

[emi470129-bib-0026] Langmead, B. , and S. L. Salzberg . 2012. “Fast Gapped‐Read Alignment With Bowtie 2.” Nature Methods 9, no. 4: 357–359. 10.1038/nmeth.1923. 22388286 PMC3322381

[emi470129-bib-0027] Liao, Y. , G. K. Smyth , and W. Shi . 2019. “The R Package Rsubread Is Easier, Faster, Cheaper and Better for Alignment and Quantification of RNA Sequencing Reads.” Nucleic Acids Research 47, no. 8: e47. 10.1093/nar/gkz114. 30783653 PMC6486549

[emi470129-bib-0028] Love, M. I. , W. Huber , and S. Anders . 2014. “Moderated Estimation of Fold Change and Dispersion for RNA‐Seq Data With DESeq2.” Genome Biology 15, no. 12: 550. 10.1186/s13059-014-0550-8. 25516281 PMC4302049

[emi470129-bib-0029] Picone, N. , S. S. Mohammadi , A. C. Waajen , et al. 2020. “More Than a Methanotroph: A Broader Substrate Spectrum for Methylacidiphilum Fumariolicum SolV.” Frontiers in Microbiology 11, no. 604485: 604485. 10.3389/fmicb.2020.604485. 33381099 PMC7768010

[emi470129-bib-0030] Pol, A. , T. R. Barends , A. Dietl , et al. 2014. “Rare Earth Metals Are Essential for Methanotrophic Life in Volcanic Mudpots.” Environmental Microbiology 16, no. 1: 255–264. 10.1111/1462-2920.12249. 24034209

[emi470129-bib-0031] Pol, A. , K. Heijmans , H. R. Harhangi , D. Tedesco , M. S. Jetten , and H. J. M. Op den Camp . 2007. “Methanotrophy Below pH 1 by a New Verrucomicrobia Species.” Nature 450, no. 7171: 874–878. 10.1038/nature06222. 18004305

[emi470129-bib-0032] Saldivar, A. , P. Ruiz‐Ruiz , S. Revah , and C. Zuñiga . 2024. “Genome‐Scale Flux Balance Analysis Reveals Redox Trade‐Offs in the Metabolism of the Thermoacidophile Under Auto‐, Hetero‐and Methanotrophic Conditions.” Frontiers in Systems Biology 4: 1291612. 10.3389/fsysb.2024.1291612. PMC12341988 40809126

[emi470129-bib-0033] Schaefer, H. , S. E. Mikaloff Fletcher , C. Veidt , et al. 2016. “A 21st‐Century Shift From Fossil‐Fuel to Biogenic Methane Emissions Indicated by 13CH4.” Science 352, no. 6281: 80–84. 10.1126/science.aad2705. 26966190

[emi470129-bib-0034] Schmitz, R. A. , S. H. Peeters , S. S. Mohammadi , et al. 2023. “Simultaneous Sulfide and Methane Oxidation by an Extremophile.” Nature Communications 14, no. 1: 2974. 10.1038/s41467-023-38699-9. PMC10205796 37221165

[emi470129-bib-0035] Schmitz, R. A. , S. H. Peeters , W. Versantvoort , et al. 2021. “Verrucomicrobial Methanotrophs: Ecophysiology of Metabolically Versatile Acidophiles.” FEMS Microbiology Reviews 45, no. 5: fuab007. 10.1093/femsre/fuab007. 33524112 PMC8498564

[emi470129-bib-0036] Seewald, J. S. 2001. “Aqueous Geochemistry of Low Molecular Weight Hydrocarbons at Elevated Temperatures and Pressures: Constraints From Mineral Buffered Laboratory Experiments.” Geochimica et Cosmochimica Acta 65, no. 10: 1641–1664. 10.1016/S0016-7037(01)00544-0.

[emi470129-bib-0037] Sharp, C. E. , A. V. Smirnova , J. M. Graham , et al. 2014. “Distribution and Diversity of Verrucomicrobia Methanotrophs in Geothermal and Acidic Environments.” Environmental Microbiology 16, no. 6: 1867–1878. 10.1111/1462-2920.12454. 24650084

[emi470129-bib-0038] Suttisansanee, U. , and J. F. Honek . 2011. “Bacterial Glyoxalase Enzymes.” Seminars in Cell & Developmental Biology 22, no. 3: 285–292. 10.1016/j.semcdb.2011.02.004. 21310258

[emi470129-bib-0039] Sutzl, L. , G. Foley , E. M. J. Gillam , M. Boden , and D. Haltrich . 2019. “The GMC Superfamily of Oxidoreductases Revisited: Analysis and Evolution of Fungal GMC Oxidoreductases.” Biotechnology for Biofuels 12: 118. 10.1186/s13068-019-1457-0. 31168323 PMC6509819

[emi470129-bib-0040] Takahashi, J. 1980. “Production of Intracellular and Extracellular Protein from n‐Butane by Pseudomonas Butanovora sp. nov.” In Advances in Applied Microbiology, edited by D. Perlman , vol. 26, 117–127. Academic Press. 10.1016/S0065-2164(08)70332-0.

[emi470129-bib-0041] van Teeseling, M. C. , A. Pol , H. R. Harhangi , et al. 2014. “Expanding the Verrucomicrobial Methanotrophic World: Description of Three Novel Species of Methylacidimicrobium Gen. Nov.” Applied and Environmental Microbiology 80, no. 21: 6782–6791. 10.1128/AEM.01838-14. 25172849 PMC4249049

[emi470129-bib-0042] Versantvoort, W. , A. Pol , L. J. Daumann , et al. 2019. “Characterization of a Novel Cytochrome c(GJ) as the Electron Acceptor of XoxF‐MDH in the Thermoacidophilic Methanotroph Methylacidiphilum Fumariolicum SolV.” Biochimica Biophysica Acta Proteins Proteomics 1867, no. 6: 595–603. 10.1016/j.bbapap.2019.04.001. 30954577

