# Supplementary material for: C3 Compound Metabolism in the Thermoacidophilic Methanotroph Methylacidiphilum fumariolicum SolV
Source: Environ Microbiol Rep. 2025 Jul 8;17(4):e70129. doi: 10.1111/1758-2229.70129 (PMC12235487; doi:10.1111/1758-2229.70129)
Supplement: Supplementary file 2 — Figure S1. | Effect of CO2 and lanthanides on the growth of M. fumariolicum SolV. Figure S2. | Propane (grey diamonds) conversion and optical density (black squares) of M. fumariolicum SolV cells initially grown on (A) 2‐propanol and (B) acetone. [file EMI4-17-e70129-s003.pdf]

Supplementary Material

C3 compound metabolism in the thermoacidophilic methanotroph Methylacidiphilum fumariolicum SolV

Changqing Liu, Arjan Pol, Stijn Peeters, Rob A. Schmitz, Theo A. van Alen, Lena J. Daumann, Huub J.M. Op den Camp* & Wouter Versantvoort

Department of Microbiology, Radboud Institute for Biological and Environmental Sciences, Faculty of Science, Radboud University Nijmegen, Nijmegen, the Netherlands.

*** Correspondence:** h.opdencamp@science.ru.nl

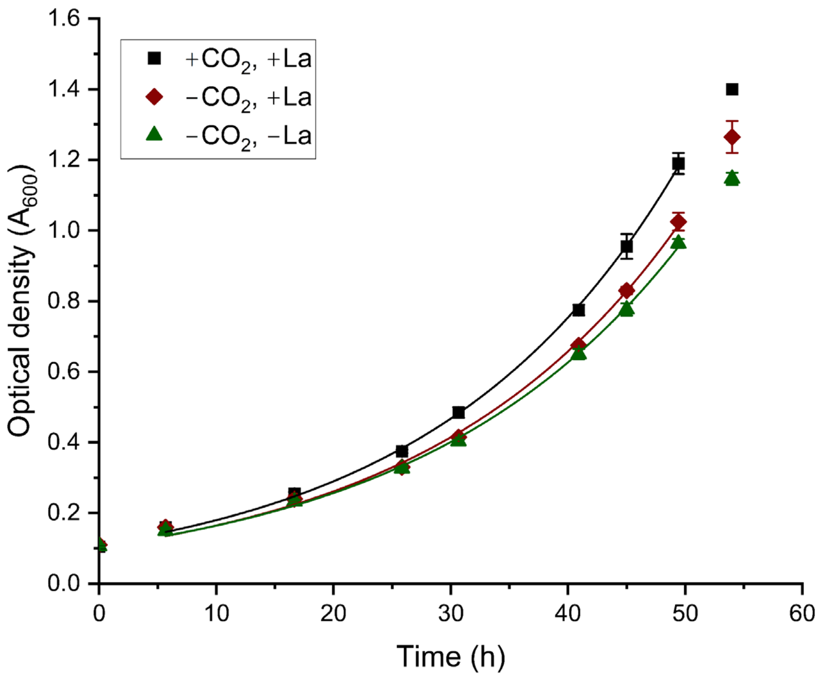

SUPPLEMENTARY FIGURE S1 | Effect of CO_2 and lanthanides on the growth of *M. fumariolicum* SolV cells on 2-propanol inoculated in medium containing 26 mM 2-propanol/5% CO_2 (black square), 2-propanol without CO_2 (red diamond) or 2-propanol without CO_2 and without lanthanides (green triangle), after an initial cultivation on 2-propanol without CO_2 and without lanthanides for 5 consecutive transfers to fresh medium with a 20 times dilution of the cells. The OD_{600} value was plotted over time and an exponential fit was performed on the exponential phase to determine the growth rates (μ). Growth rates were 0.045, 0.040 and 0.041 h^{-1} for 5% CO_2, no CO_2 and no CO_2 & no Ln, respectively. Data are represented as mean ± SD (n=3)

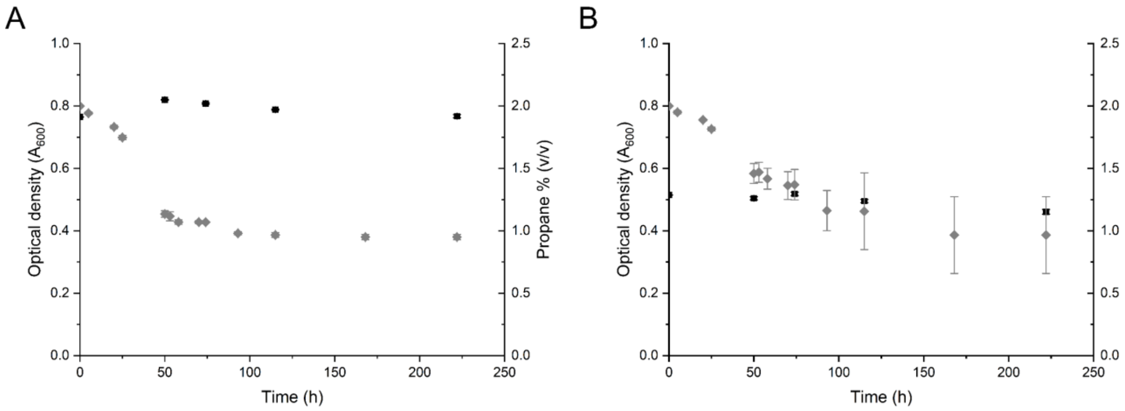

SUPPLEMENTARY FIGURE S2 | Propane (grey diamonds) conversion and optical density (black squares) of *M. fumariolicum* SolV cells initially grown on **(A)** 2-propanol and **(B)** acetone. Propane was converted by these cultures, but no concomitant increase in OD_{600} values was observed, showing that although propane was converted, growth on propane as a sole energy source was not possible. Data are represented as mean ± SD (n=3).
